# Assessing community health workers’ time allocation for a cervical cancer screening and treatment intervention in Malawi: a time and motion study

**DOI:** 10.1186/s12913-022-08577-z

**Published:** 2022-09-23

**Authors:** Jobiba Chinkhumba, Dorothy Low, Evelyn Ziphondo, Lizzie Msowoya, Darcy Rao, Jennifer S. Smith, Erik Schouten, Victor Mwapasa, Luis Gadama, Ruanne Barnabas, Lameck Chinula, Jennifer H. Tang

**Affiliations:** 1 Department of Health Systems and Policy, Kamuzu University of Health Sciences, Blantyre, Malawi; 2 Health Economics and Policy Unit, Kamuzu University of Health Sciences, Blantyre, Malawi; 3 Department of Global Health, University of Washington, Seattle, USA; 4 UNC Project, Lilongwe, Malawi; 5 Department of Epidemiology, University of Washington, Seattle, USA; 6 University of North Carolina at Chapel Hill, Chapel Hill, NC USA; 7 Lineberger Comprehensive Cancer Center, Chapel Hill, NC USA; 8 Division of Infectious Diseases, Massachusetts General Hospital, Boston, MA USA; 9 Harvard Medical School, Boston, MA USA; 10 Department of OB-GYN, University of North Carolina at Chapel Hill, Chapel Hill, NC USA

**Keywords:** Community health workers, Cervical cancer screening and prevention therapy, Time and motion study

## Abstract

**Background:**

Community health workers (CHWs) are essential field-based personnel and increasingly used to deliver priority interventions to achieve universal health coverage. Existing literature allude to the potential for detrimental effects of multi-tasking CHWs. This study objective was to assess the impact of integrating cervical cancer screening and prevention therapy (CCSPT) with family planning (FP) on time utilization among CHWs.

**Methods:**

A time and motion study was conducted in 7 health facilities in Malawi. Data was collected at baseline between October-July 2019, and 12 months after CCSPT implementation between July and August 2021. CHWs trained to deliver CCSPT were continuously observed in real time while their activities were timed by independent observers. We used paired sample t-test to assess pre-post differences in average hours CHWs spent on the following key activities, before and after CCSPT implementation: clinical and preventive care; administration; FP; and non-work-related tasks. Regression models were used to ascertain impact of CCSPT on average durations CHWs spent on key activities.

**Results:**

Thirty-seven (*n* = 37) CHWs were observed. Their mean age and years of experience were 42 and 17, respectively. Overall, CHWs were observed for 323 hours (inter quartile range: 2.8–5.5). Compared with the period before CCSPT, the proportion of hours CHWs spent on clinical and preventive care, administration and non-work-related activities were reduced by 13.7, 8.7 and 34.6%, respectively. CHWs spent 75% more time on FP services after CCSPT integration relative to the period before CCSPT. The provision of CCSPT resulted in less time that CHWs devoted towards clinical and preventive care but this reduction was not significant. Following CCPST, CHWs spent significantly few hours on non-work-related activities.

**Conclusion:**

Introduction of CCSPT was not very detrimental to pre-existing community services. CHWs managed their time ensuring additional efforts required for CCSPT were not at the expense of essential activities. The programming and policy implications are that multi-tasking CHWs with CCSPT will not have substantial opportunity costs.

**Supplementary Information:**

The online version contains supplementary material available at 10.1186/s12913-022-08577-z.

## What is already known on this topic

Community health workers are essential field-based personnel who are increasingly used to deliver priority maternal and child health interventions in resource poor settings.

Existing literature allude to potential detrimental effects of multi-tasking CHWs but fail to meaningfully assess the extent to which this is true/feasible.

## What this study adds

This study is among the first to evaluate the impact of integrating a cervical cancer screening and prevention therapy intervention with Family Planning on CHWs time utilization. Comparative analysis indicates that the existing task environment can readily accommodate new community interventions such as cervical cancer screening and prevention therapy.

## What do the new findings imply?

It is feasible for CHWs to take on additional health interventions without causing substantial unintended consequences to existing community-based health programs or services.

Therefore, it is important to continue exploring opportunities to leverage CHWs to support global efforts towards elimination of cervical cancer.

## Introduction

Globally, there is recognition that involving community health workers (CHWs) in the delivery of priority maternal and child health services is essential to achieving the goal of Universal Health Coverage [1, 2]. In limited resource settings facing human resource constraints for meeting the dual burden of infectious and chronic diseases [3], investments are being made in CHW programs to expand CHWs utilization and meet population healthcare needs, reduce health inequities and make services more accessible [4, 5].

CHWs are community-level practitioners selected from peer community members. CHWs play a critical role by connecting the communities with the formal healthcare sector [3]. They typically provide primary healthcare to residents with an emphasis on preventive services [6]. In this regard, CHWs function as an initial point of care by meeting people “where they are” and interacting with hard-to-reach individuals at the household level.

Within the context of maternal health, the current scope of CHWs service portfolio includes health education, breastfeeding promotion, essential newborn care, psychosocial support and family planning services [7]. Substantial evidence support the positive impact of CHWs on disease prevention, health promotion and improved access to healthcare in diverse contexts [8, 9]. Building on this success, program implementers continue to expand the scope of CHWs to respond to emerging health system needs, including improving access to care for adults living with chronic conditions [10, 11]. Given the potential opportunity costs associated with augmenting the CHWs workload with additional responsibilities, it is important to consider the effects that new tasks may have on CHWs performance and service provision [12–14]. Therefore, decisions regarding the expansion of CHWs workload should weigh the anticipated benefits of new responsibilities, while simultaneously preserving the volume and quality of existing CHWs essential service provision. In this regard, the design and implementation of novel community-based reproductive health interventions to increase population coverage should be informed by context-specific evidence of CHWs performance expectations and staffing norms [15]. However, it remains unclear how new community-based interventions influence CHWs time use. Considering the increasing international interest in leveraging the CHWs cadre to strengthen health systems responses [7], a better conceptualization of CHWs resource allocation will provide critical insights for global health policy and reproductive health programming.

We evaluated the impact of integrating a cervical cancer screening and prevention therapy (CCSPT) intervention with Family Planning (FP) on CHWs time utilization [16]. Addressing impact of such novel approaches has important implications for Malawi and other low-income countries working to scale up cervical cancer screening and treatment. The Malawi Ministry of Health (MoH) National Cervical Cancer Control Strategy has the explicit target of screening for the first time 80% of women aged 25–49 years [17]. We sought to describe CHWs work tasks and identify opportunities for workload expansion. Our primary objectives were: i) to describe CHWs time utilization before and after CCSPT intervention, and ii) to quantify CHW time allocation for both work and non-work activities before and after the CCSPT intervention.

## Methodology

Malawi, with 19 million inhabitants, has the second highest cervical cancer incidence and mortality in the world with age standardized rate of 75.9 and 49.8 per 100,000, respectively [18, 19]. About 27% of Malawian women receive cervical cancer screening and only 43% with positive results receive care. HIV prevalence among women 15–49 years is 8.8% [20]. In 2021, Malawi had 0.14 doctors and 0.33 nurses and midwives per 1,000 people [21]. This critical shortage of health workers makes it difficult to increase CCSPT services, particularly in rural areas where there are few providers. CHWs (known as health surveillance assistants in Malawi) may help to address shortage challenges as this cadre is increasingly leveraged to address service provision bottlenecks. CHWs are salaried and employed by the Ministry of Health [22]. They provide their services through Outreach and Village Clinics. Ideally, one CHW should cater for 1,000 community members, but as of 2021, the ratio was 0.55 CHW per 1,000 people [21].

This study was done at 7 health facilities in Lilongwe and Zomba, two of Malawi’s 28 districts serving a population of about 3.8 million [18]. The 7 health facilities were selected because they were all randomized to implement a prevention of cervical cancer through a human papillomavirus (HPV)-based screen and treat intervention with a community component involving CHWs.

### Cervical cancer screening and prevention therapy

Details of the CCSPT intervention have been provided elsewhere [16]. Briefly, the intervention integrates HPV-based CCSPT into family planning (FP) services with two models of care. In facilities randomized to model 1 (clinic-only), self-sampling for HPV is offered to women 25 to 49 years with no history of total hysterectomy attending FP services at health facilities. Same-day thermal ablation is provided to HPV-positive, ablation-eligible women. In facilities randomized to model 2 (clinic + community), screening is offered as in model 1 and in addition, community-based screening is also offered to eligible women by CHWs. The CHWs transport collected samples for HPV testing to health facilities, collect HPV results and refer HPV-positive women to the nearest health facility for treatment.

### Study design

We conducted an observational time and motion study to describe the work activities undertaken by CHWs at the 7 model 2 facilities. Independent data collectors followed CHWs throughout the workday and performed continuous direct observations of both clinical and non-clinical activities in which the providers engaged [23]. We used a pre/post approach to assess the impact of CCSPT on CHWs time use. The CHWs providing community based CSSPT were each observed twice. Baseline data collection occurred from October to November 2019 prior to CCSPT implementation. Endline data collection occurred from July to August 2021, one year following the roll out of the CCSPT intervention, when the health facilities were judged to be in a steady state of routine CCSPT service provision.

### Study eligibility

CHWs who had undergone CCSPT training and were providing services in model 2 facility catchment areas were invited to participate. Trainee CHWs were excluded from the study since they did not have Village Clinics and would thus not be accessed by eligible women.

We recruited CHWs who agreed to participate by giving verbal informed consent. Once recruited before the CCSPT implementation, we aimed to observe the same CHWs after the CCSPT implementation. To minimise the Hawthorne effect, or the change in some aspect of the observed behaviour due to awareness of being observed [24], each CHW was observed twice during each period. The first observation session was a simulation, meant to allow the CHWs to habituate to being observed and facilitate more natural behaviours during official data collection. The second session was for actual data collection. We used data from the second sessions only for analyses. CHWs were not aware that only data from the second observations would be used for data analysis. CHWs showed some awareness of being observed during the first observations as they made effort to explain to observers what were about to do/ doing or sometimes attempted to initiate conversations with the observers. In such cases, observers were instructed to politely remind the CHWs to ignore them (observers) and focus on their tasks. Hardly any of such CHWs behaviours were noted during the 2^nd^ observations.

### Observers’ training

Ten research assistants served as observers, none of whom were CHWs themselves. They all underwent a two-day classroom-based training in time and motion studies led by an experienced trainer (JC). The specific roles and activities of CHWs in Malawi are outlined in a national guideline [22]. This informed the development of time and motion activities. During the training, the observers studied these main activities and corresponding sub-activities (Appendix 1). They were instructed by an experienced Community Nurse (EZ) to identify the start and end of each sub-activity without the need to ask the observed CHWs what they were doing. The research assistants were also instructed on how to collect data using tablet computers. A one-day pilot session in which the research assistants practiced observations on non-study CHWs was conducted after the classroom-based training. The observers were encouraged to ask questions to the observed or instructors for clarifications during the pilot session. Experiences and lessons learnt from the pilot were used to modify and improve sub-activity definitions and grouping of activities including refining the structure of the data collection tool.

### Main activities, sub-activities and analysis groups

A pre-defined tool composed of a set of activities logically organized to facilitate data collection and analysis was used to document CHWs activities (Appendix 1). CHWs tasks were categorised into 7 main activities: 1) Under 5 children treatment services; 2) Under 5 children preventive services; 3) FP services; 4) CCSPT services; 5) Over 5 services; 6) Administration; and 7) Non-work-related tasks. Each of the main activities had corresponding sub-activities (Appendix 1). Because the CCSPT period coincided with the COVID-19 pandemic, the endline data collection tool was modified to include a COVID-19 sub-activity. We structured the sub-activities so that they could easily be visually identified when each started and ended without the need for CHWs to explain what they were doing. This structure was crucial to the success of the data collection because the observer’s role during data collection was passive involving no communications with the observed.

For analysis, the sub-activities were further collapsed into five groups: 1) Clinical and preventive services; 2) FP; 3) CCSPT; 4) administration and 5) non-work-related tasks. Clinical and preventive services included the whole spectrum of curative and preventive services offered to children < 5 years of age, such as history taking, testing and treatment for acute illnesses, provision of immunizations and growth monitoring. This task grouping also included sub-activities provided to those ≥ 5 years such mass administration of deworming drugs to school age children and provision of diagnostic services to adults for infectious diseases such as tuberculosis and HIV including making appropriate referrals to health facilities. FP services included providing health education to women on FP options and delivery of preferred methods. CCSPT services included counselling about self-sampling for HPV, transporting samples to facilities for testing, delivering HPV results to women and making referrals. Administrative tasks consisted of general duties such staff meetings, official travels, supervisions and report writings. The non-work-related task group consisted of all informal activities, including making personal calls, staying idle, chatting with other health workers and sending SMS or WhatsApp texts for personal or non-work-related purposes.

### Data collection

**Fig. 1:**
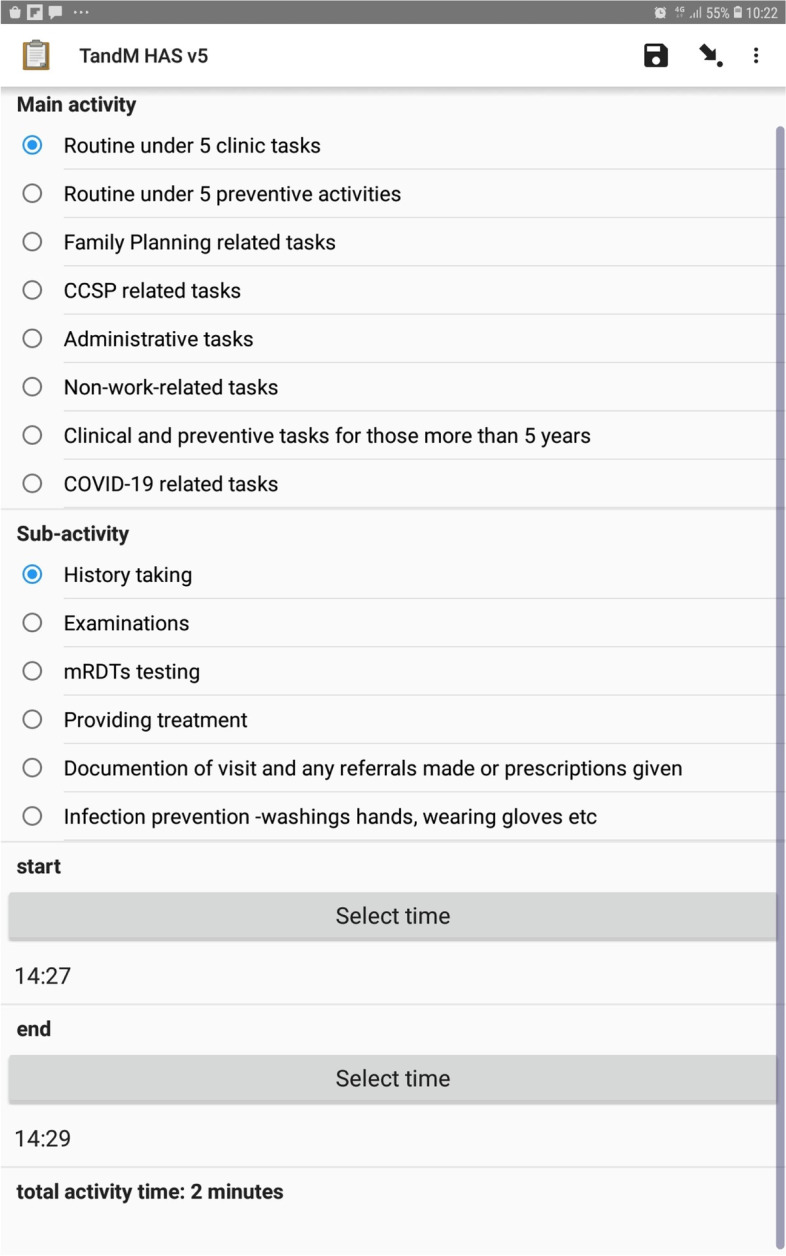
Screen shot of the data collection tool To log a sub-activity, the observer had to first identify the main activity under which the sub-activity was listed, then select the main activity. Drop down list of sub-activities would then appear from which the observer would select the sub-activity of interest. A tap on the start and end buttons initiated the start and end of a sub-activity timing, respectively

We used a structured data collection instrument (Appendix 1) programmed digitally with Open Data Kit software to facilitate electronic data capture using Samsung Galaxy-Tab-2.0 tablets [25]. Each main activity group appeared as a menu. Thus, to log a sub-activity, the observer had to first identify the main activity under which the sub-activity was listed, then select the main activity, and finally select the sub-activity of interest (Fig. 1). A tap on the start and end buttons by the observer initiated the start and end of a sub-activity timing, respectively. The sub-activity durations were automatically timed by internal clock of the tablet computers between tapping of the start and end buttons. The tablet was also used to capture additional information about the CHWs being observed including their age, sex, years of work experience, catchment population and the name of the nearest health facility.

Only one sub-activity at a time could be captured by the tablet computers. In cases where the CHWs were performing more than one task simultaneously (rare events), for example, examining a sick child while talking to a colleague, it was up to the observer to decide which sub-activity was the dominant one to be timed. For some sub-activities, CHWs often switched between tasks or sub-activities, for instance administering vaccines to children, chatting with a colleague, going back to vaccine administration and then chatting on WhatsApp. The tool was flexible enough to accurately capture data for such sequence of fleeting sub-activities. But the tool was unable to collate tasks that were interrupted by other tasks. For example, if a CHW was engaged in history taking with Patient A, then briefly left to provide contraception to Patient B, and finally returned to complete history taking with Patient A, the data collection system would register the provider’s actions as three distinct tasks (i.e., two tasks of history taking and one task of contraception provision). Thus, activities were observed in singularity. The data collection system did not allow for activity concurrency (i.e., multi-tasking). At the end of each day, data were backed up in the computer tablets and uploaded to a central server for quality control and safe storage.

### Data analysis

The primary outcome of interest was the average time in hours spent by a CHW providing routine clinical and preventive services during an average day after the CCSPT implementation. The rationale for choosing this outcome was to ascertain effects of CCSPT on traditional CHW curative and preventive services. Secondary outcomes of interest were time in hours spent providing FP, CCSPT, administrative duties and doing non-work-related activities per CHW. The time spent on each outcome was estimated by adding up all sub-activity durations in minutes under its respective analysis group (clinical and preventive services, FP, CCSPT, administration, and non-work-related tasks) during the observation period for each CHW and then dividing the total by 60.

We conducted descriptive statistics, mean estimates for each outcome and associated 95% confidence intervals. We used paired sample t-test to ascertain mean differences between groups before and after CCSPT. We defined statistical significance as a *p* value < 0.05. For each outcome whose mean significantly changed after CCSPT in these crude analyses, we evaluated the change in time after CCSPT using multivariable repeated measures mixed regression models. This approach accounted for the correlation between observations contributed by each CHW in both the pre- and post-intervention periods [26]. The main independent variable in the models was CCSPT, an indicator variable coded 1 if the observation was during CCSPT implementation and 0 otherwise. Other control variables included age, sex, years of work experience, and facility catchment area population, selected based on existing literature on determinants of CHWs performance or motivations [27, 28]. Given the small number of CHWs observed, we bootstrapped the results based on clustering at the CHW level to estimate parameter standard errors and corresponding confidence intervals [29].

### Ethical approval and consent to participate

Ethical approval for the study was provided by National Health Sciences Research Committee (NHSRC) protocol number 19/03/2355 and University of North Carolina (UNC) Institutional Review Board (IRB) protocol number 21094. Verbal informed consent was obtained from all Community Health Workers before start of data collection. The procedure for obtaining verbal informed consent from the participants was ethically approved by both the NHSRC and UNC IRBs. All methods were carried out in accordance with relevant guidelines and regulations.

## Results

The characteristics of the 7 study health facilities are shown in Table 1. The average numbers of catchment area population, FP providers and CHWs per health facility were 96,773, 16 and 19, respectively. The average ratio of CHW to catchment area population was 1 per 5,231 and ranged from 1,140 to 15,682.

**Table 1:** Characteristic of 7 participating health facilities

**Attribute**	**Mean**	**Range**
Catchment area population	96,773	30,767–324,000
Number of FP^a^ providers	16	7–22
Number of Village Clinics	24	7–39
Number of CHWs^b^	19	6–27
Population / CHW ratio	5,231	1,140–15,682

^a^ Family Planning

^b^ Community Health Workers

### Baseline community health workers’ characteristics

In total, 81 CHWs were observed at baseline before CCSPT. Of these, 37 (45.6%) were also observed after CCSPT implementation. We could not observe 44 CHWs during follow up because most 32 (72.7%) had been assigned to provide COVID-19 vaccines at health facilities, the remainder had either been transferred or were attending trainings. The rest of our analyses are limited to the 37 CHWs who were observed at both baseline and endline. At baseline, the average age of the CHWs was 42 years, 35% were females and their mean years of community work experience was 17 years (Table 2).

**Table 2:** Socio-demographic features of 37 community health workers

Attribute	Mean	Range or (95% CI^a^)
Age (in years)	42	32–54
Sex (% females)	35	21-52^a^
Years of experience	17	(8–27)

^a^ 95% confidence interval

### The relative distribution of community health workers’ time by activity group, before and after CCSPT

The share of time CHWs committed towards non-CCSPT clinical and preventive care, administration and non-work related-tasks were 13.7, 8.7 and 34.6% lower after CCSPT was implemented compared with the period before CCSPT, respectively. CHWs devoted 75% more time to FP services after CCSPT implementation relative to the period before implementation (11.9% vs 6.8%). CHW spent 11.4% of their overall time on CCSPT-related activities, which was largely at the expense of non-work-related tasks as shown in Table 3.

**Table 3:** Community Health Workers’ time allocation towards main activities, before and after CCSPT

**Main activity**	**Before CCSPT**	**After CCSPT**	**Difference**
	**%**	**%**	**%**
Clinical and preventive tasks	31.4	27.1	**-13.7**
Administration tasks	35.5	32.4	**-8.7**
Family planning tasks	6.8	11.9	**75.0**
CCSPT tasks		11.4	
Non-work related-tasks	26.3	17.2	**-34.6**
**Overall**	**100**	**100**	

*CCSPT* Cervical Cancer Screening and Prevention Therapy

### Community health workers’ average time use in hours by activity group, before and after CCSPT

We conducted 323.0 (IQR 2.8 to 5.5) hours of direct time and motion observations for the 37 CHWs. About 175.5 (IQR 3.4 to 6.0) hours of observations were conducted at baseline before CCSPT implementation, compared with 147.4 (IQR2.7 to 4.2) hours of observation at endline.

On average, CHW spent more hours per day at Village Clinic before CCSPT, 4.74 compared with 3.98 after CCSPT, *p* = *0.04*. The amount of time CHWs devoted towards administrative and FP tasks was not substantially different before and after CCSPT; however, CHWs spent significantly less time on clinical and preventive care 1.21 vs 1.53 h: *p* = *0.03* and non-clinical tasks: 0.77 vs 1.28 h, *p* = *0.02* during the CCSPT period compared with the period before CCSPT. CHWs spent on average 0.51 hours per day (range: 0.02 to 3.13) providing CCSPT services to women (Table 4).

**Table 4:** Average time (hours) Community Health Workers spent per activity group, before and after CCSPT

		**Before CCSPT**		**After CCSPT**		
**Activity group**	**N**	**Mean**	**95% CI**	**Mean**	**95% CI**	***p value***
Clinical and preventive care	35	1.53	1.24–1.82	1.21	0.92 -1.50	0.03
Administration	37	1.73	1.23–2.24	1.45	1.18–1.71	0.33
Family planning	13	0.33	0.16–0.52	0.53	0.28–0.79	0.92
CCSPT tasks	26			0.51	0.02–3.13^*^	NA
Non-work-related tasks	34	1.28	0.88–1.69	0.77	0.55–1.01	0.02
**Overall time in hours**	**37**	**4.74**	**4.06–5.42**	**3.98**	**3.42–4.55**	**0.04**

^*^ Range not 95% CI

*NA* Not applicable

Table 5 shows the impact of CCSPT on the average time CHWs spent on clinical and preventive care (model A) and non-work-related activities (Model B), adjusted for control variables. CCSPT was associated with a reduction in average time CHWs devoted towards clinical and preventive care per day but this was not statistically significant. CCPST was significantly associated with a reduction in amount of time CHWs spent on non-work-related tasks per day. None of the control variables had significant influence on the mean time CHWs spent on clinical and preventive care or non- work-related tasks.

**Table 5:** Effects of CCSPT intervention on Community Health Workers average time on clinical and non-work-related tasks, adjusted for control variables

**Dependent variable**		**Model A**			
Average hours spent on clinical and preventive tasks/ CHW					
		***Bootstrap***			
**Covariates**	***Coef***	***Std. Err***	***P value***	***95% CI***	
CCSPT	-0.27	0.23	0.24	-0.72 to 0.18	
Age	0.05	0.04	0.26	-0.03 to 0.12	
Sex	0.05	0.21	0.81	-0.36 to 0.46	
Years of experience	-0.03	0.05	0.53	-0.12 to 0.06	
Population	-0.47	1.66	0.78	-3.72 to 2.77	
**Dependent variable**		**Model B**			
Average hours spent on non-work-related tasks / CHW					
		***Bootstrap***			
**Covariates**	***Coef***	***Std. Err***	***P value***	***95% CI***	
CCSPT	-0.73	0.29	0.01	-1.45 to -0.15	
Age	-0.02	0.04	0.73	-0.10 to 0.07	
Sex	-0.27	0.25	0.28	-0.75 to 0.21	
Years of experience	0.06	0.04	0.15	-0.02 to 0.15	
Population	-0.01	1.54	1.00	-3.02 to 3.00	

*Coef* Coefficient, *CHW* Community health worker, *CCSPT* Cervical Cancer Screening and Preventive Therapy, *95% CI* 95% Confidence interval, *Std.Err* Standard error

## Discussion

Multi-tasking of CHWs to increase population coverage with innovative community-based interventions may come at a cost to core CHWs services such as delivery of expanded program of immunisation and integrated management of childhood illness [12]. We tested this hypothesis by assessing the impact of CCSPT on CHWs time use. The implementation of CCSPT was associated with a 14% decrease in the share of time CHWs devote towards clinical and preventive services. As currently designed and implemented, CCSPT appears in the short term not to be very detrimental to routine clinical and preventives services. However, further studies should estimate the health benefits foregone from displaced services (in children and adults) and health benefits gained from CCSPT when CHWs time is re-allocated from the former to the latter. If the health gains are greater than those foregone, there would be a net gain at the broader health systems level justifying CHWs time re-allocations, although such health benefits redistribution may raise equity concerns.

The introduction of CCSPT was associated with a 75% increase in the amount of time CHWs devoted towards FP services, demonstrating synergy when CCSPT is integrated with FP services. While increasing volume of FP services is desirable given current FP unmet needs in Malawi [30] and other low and middle income countries [31], future studies should ascertain quality of both CCSPT and FP care following integration to ensure volume increase does not come at the expense of quality.

The study found that CHWs spend 26.3% of their time (1.28 hours/day) on non-work-related activities. These results are inconsistent with current perceptions that CHWs are over burden with work-related-tasks [5]. The fact that the amount of time CHWs spend on non-work-related tasks was substantially reduced post CCSPT implementation highlights CHWs potential to absorb additional formal tasks and their capacity to rationalize time use by substituting informal with formal activities when faced with new work demands. Thus, CHWs should receive regular supervisory support field visits and incentivized to ensure optimal use of their time [32].

The average time a CHW spends working per day is 4.7 hours, accounting for 58.8% of the official 8 hour per day schedule [33]. Again, this gap underscores capacities for CHWs to take on additional health interventions without undermining current activities as long as they can manage their time optimally. Whether CHWs in the country can be nudged to work more hours within the 8 hour/day ceiling is contingent on several factors including local work policies and how motivated CHWs are [5, 28]. Therefore, facility managers should explore innovative ways to increase CHW efficiency and performance. Importantly, CHW should be mentored and supported to improve time allocation within and across health programs. Moving forward, we argue for close collaboration between the Reproductive Health Directorate (responsible for FP services) and Community Health department (responsible for CHWs) to ensure that roll out of new programs such as CCSPT has minimal un-intended consequences.

The programming and policy implication of these findings in our setting are that tasking CHW with CCSPT-related tasks may not have large opportunity costs and that CHWs should continue playing primary roles in the delivery of community-based CCSPT to increase universal health coverage (UHC). This finding is important for Malawi as it is among the World Health Organization Member States that have endorsed the global strategy towards the elimination of cervical cancer, aiming to screen 70% of women with high-performance tests and appropriately treating 90% of those identified with cervical dysplasia or cancer [34].

The strengths of this study are based on the use of time and motion approach, a more reliable means to quantify time utilization and ascertaining how time is allocated to different types of activities compared to alternative approaches [35]. The use of same study participants before and after exposure minimised biases that would have occurred if different participants were observed between the observations. Finally, this study provides timely evaluation of CHWs allocation since more interest is being paid leveraging CHWs to address unmet health needs in limited resource settings. Nonetheless, this study has some limitations. First, there was high attrition (54%). However, the composition of the study participants and those that could not be followed up in terms of age, sex and years of experience did not differ substantially. We thus believe that the results are generally representative of CHWs in our settings. Secondly, another limitation of this study is that CHWs were not interviewed as part of the Time-and-Motion study to provide feedback about the integration of cervical cancer screening and treatment activities. Their voice would have been helpful in contextualizing our results. Finally, although CHWs can conduct two tasks simultaneously, we could only record one activity at a time. Though such scenarios were rare, such omissions can underestimate time CHWs spend on activities. To overcome this limitation, future studies should assess activity concurrency which would allow estimates of efficiencies or time savings from multi-tasking.

## Conclusion

This study presents estimates for CHWs time allocation to provide a basis for engaging in discussions and planning about expansion of CHWs workload within the context of increasing UHC for priority health interventions. There is scope for CHWs to take on additional health interventions without causing substantial un-intended consequences to pre-existing programs. However, close supervision of CHWs to improve their time management within and across health interventions and close collaboration between health programs is essential for overall community health systems service delivery and performance as policy makers and programmers expand CHWs work portfolios with new programs.

## Supplementary Information

**Additional file 1: Appendix 1.**

## Data Availability

Data is available upon request to the corresponding author.

## Abbreviations

CCSPT: Cervical Cancer Screening and Prevention Therapy
CHW: Community Health Worker
CI: Confidence Interval
FP: Family Planning
HF: Health Facility
IRB: Institutional Review Board
IQR: Inter Quartile Range
NHSRC: National Health Sciences Research Committee
ODK: Open Data Kit
UHC: Universal Health Coverage
UNC: University of North Carolina
VC: Village Clinic
